# Frequency Modulation and Absorption Improvement of THz Micro-bolometer with Micro-bridge Structure by Spiral-Type Antennas

**DOI:** 10.1186/s11671-018-2484-7

**Published:** 2018-03-05

**Authors:** Jun Gou, Qingchen Niu, Kai Liang, Jun Wang, Yadong Jiang

**Affiliations:** 1 State Key Laboratory of Electronic Thin Films and Integrated Devices, University of Electronic Science and Technology of China, Chengdu, 610054 China; 2 School of Optoelectronic Information, University of Electronic Science and Technology of China, Chengdu, 610054 China

**Keywords:** THz, Micro-bolometer, Spiral-type antenna, Absorption, Frequency modulation

## Abstract

Antenna-coupled micro-bridge structure is proven to be a good solution to extend infrared micro-bolometer technology for THz application. Spiral-type antennas are proposed in 25 μm × 25 μm micro-bridge structure with a single separate linear antenna, two separate linear antennas, or two connected linear antennas on the bridge legs, in addition to traditional spiral-type antenna on the support layer. The effects of structural parameters of each antenna on THz absorption of micro-bridge structure are discussed for optimized absorption of 2.52 THz wave radiated by far infrared CO_2_ lasers. The design of spiral-type antenna with two separate linear antennas for wide absorption peak and spiral-type antenna with two connected linear antennas for relatively stable absorption are good candidates for high absorption at low absorption frequency with a rotation angle of 360*n (*n* = 1.6). Spiral-type antenna with extended legs also provides a highly integrated micro-bridge structure with fast response and a highly compatible, process-simplified way to realize the structure. This research demonstrates the design of several spiral-type antenna-coupled micro-bridge structures and provides preferred schemes for potential device applications in room temperature sensing and real-time imaging.

## Background

Terahertz (THz) radiation (0.1~10 THz, 1 THz = 10^12^ Hz), proven to have unique spectral characteristics of wide band, low energy penetration and spectral absorption [1, 2], is attractive for its wide variety of applications in molecular spectroscopy [3], disease diagnostics [4], sensing, and imaging [5, 6]. However, this frequency range has not been fully exploited to date, restricted by the dearth of THz-tuned sources and detectors. In the past 20 years, the developments of ultrafast electronics, laser technology, and low-scale semiconductor technology have provided effective ways for THz wave emission and detection. Quantum cascade lasers (QCL) can radiate line emission at tunable frequencies [7, 8] while far infrared CO_2_ gas laser emitting 2.52 THz wave provides much higher radiant power [9]. Currently, THz detectors are mainly based on two kinds of effects that can directly measure THz signals: photon effect and photothermal effect. Photon detector works based on the photoelectric effect of the absorbed THz radiation, including superconductor–insulator–superconductor tunnel junction (SIS) [10] and quantum well (QW) detectors working in photoconductive or photovoltaic mode [11–14]. Photon detectors have a high sensitivity and a short response time, but they are selective in wavelength and often require refrigeration. Photothermal detectors, such as room temperature pyroelectric detectors [15] and micro-bolometers [8, 9], absorb the energy of THz radiation and convert it to resistivity or spontaneous polarization changes of the thermal sensitive films. A micro-bolometer detector can be operated at room temperature with a broad wavelength response and has great advantages in array integration and cost compared to pyroelectric detectors. The development of THz micro-bolometer detector benefits from mature infrared (IR) micro-bolometer technology with the same thermal conversion mechanism. More recently, theoretical research and experimental verification of THz sensing and imaging systems have been reported based on IR micro-bolometer focal plane arrays (FPA) equipped with proper illuminating sources [7, 16]. However, such IR detectors with traditional micro-bridge structures have a low sensitivity in THz range due to poor absorption of THz radiation [17].

Some improvements have been made for enhanced THz absorption of traditional micro-bolometer micro-bridge structure. Impedance matching metal thin film, proven to absorb THz wave due to resistive loss, is the first choice as an absorbing layer in micro-bridge structures for its low heat capacity, high thermal conductivity, and good compatibility with the fabrication process of THz micro-bolometers [18, 19]. The absorption of metal thin film can be further improved by preparation process control and surface modification [20]. However, the absorption effect of single metal thin film is limited with an ideal absorption rate of 50% [21]. Metamaterial absorber and antenna tuned to the illuminator frequency can be integrated in bolometers for high absorption due to ohmic loss and dielectric loss in the structure [22, 23]. Antenna-coupled micro-bridge structure is proven to be a more effective way to achieve high absorption and sensitivity for its better compatibility in integration with micro-bolometers. The antenna provides high absorption of THz wave, while the micro-bridge structure ensures high performance thermal detection. Antenna-coupled vanadium oxide (VO_x_) thin film bolometer working at 94 GHz [24] and antenna-coupled metal-oxide-semiconductor FET (MOSFET) micro-bolometer sensitive for 0.5~1.5 THz [25, 26] are reported. Real-time imaging at 2.5 THz has been developed by CEA-Leti using antenna-coupled micro-bolometer FPAs with a QCL as THz radiation source [27]. In most cases, planar antenna structures are adopted for a large absorption area and simple fabrication process. However, wire antennas with a smaller bulk volume are preferable over planar antennas for a faster heating rate which lead to a lower thermal response time [28].

In our earlier research [29], spiral-type wire antenna was introduced in 35 μm × 35 μm micro-bolometer micro-bridge structure, and a new type of spiral antenna with extended legs was put forward preliminarily for improved absorption of 2.52 THz wave. However, optimized design of antenna structure and detailed discussions on its characteristics of THz absorption, photothermal effect, and fabrication process have not been achieved. In this paper, based on micro-bridge structure with a much smaller size of 25 μm × 25 μm, three types of spiral-type antennas are proposed for THz absorption enhancement and absorption frequency modulation with a single separate linear antenna, two separate linear antennas, or two connected linear antennas on the bridge legs, in addition to traditional spiral-type antenna on the support layer. By optimization of structural parameters and analysis of absorption characteristic for each type of antenna, preferred schemes of antenna-coupled micro-bridge structures are obtained for wide absorption peak near 2.52 THz or stable absorption at 2.52 THz with high integration, simplified fabrication process, and fast heating rate.

## Results and Discussion

**Fig. 1:**
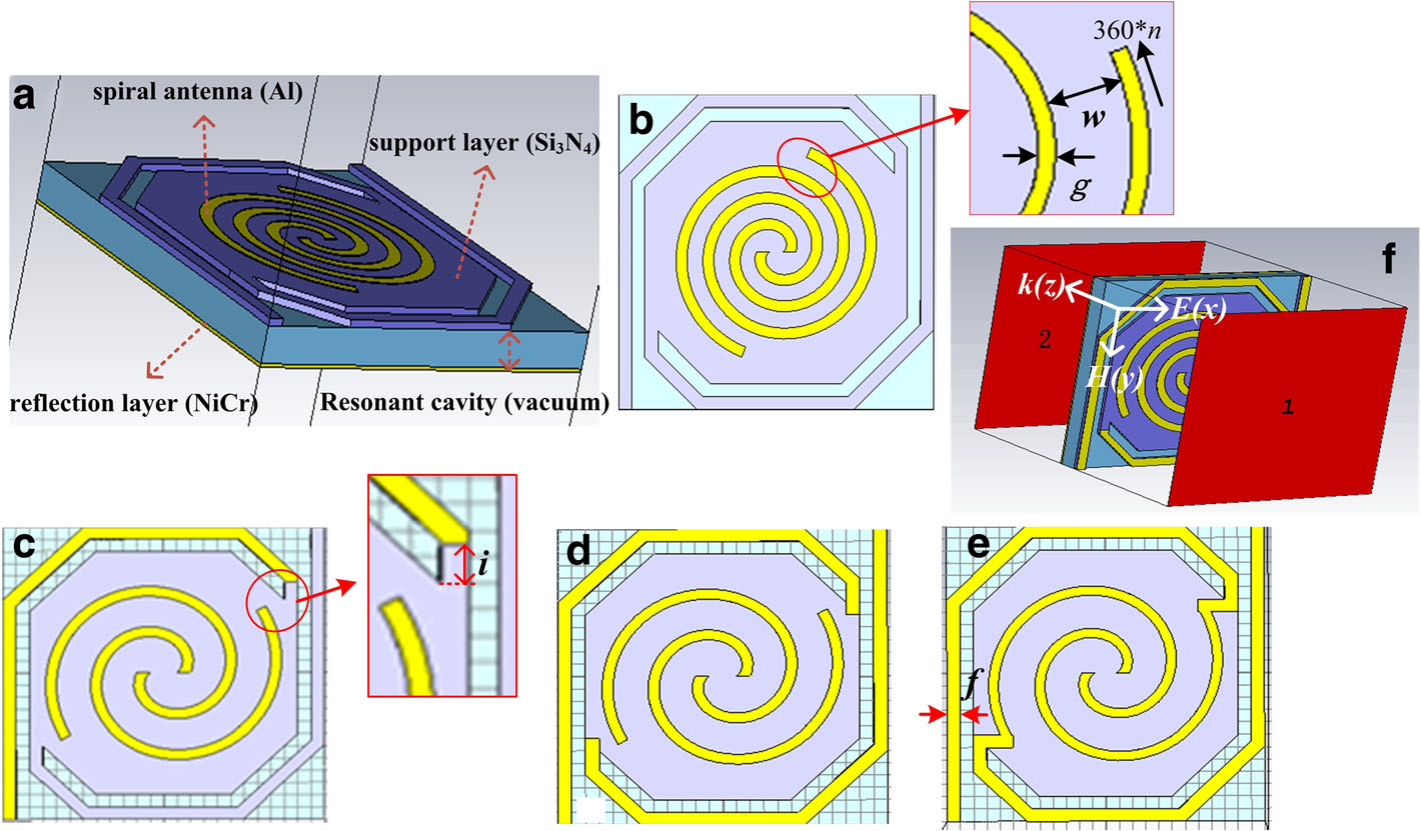
Design of spiral-type antenna-coupled micro-bridge structures. **a** Model of micro-bridge structure. **b** Spiral-type antenna on the support layer. **c** Spiral-type antenna with a single separate linear antenna on one of the bridge legs. **d** Spiral-type antenna with two separate linear antennas on the bridge legs. **e** Spiral-type antenna with two connected linear antennas on the bridge legs. **f** Directions of electric field and magnetic field for vertical incident light

The spiral-type antennas were designed for absorption enhancement and modulation of THz micro-bolometer FPAs based on micro-bridge structures with a target frequency of 2.52 THz. A single pixel in the FPAs with a pixel spacing of 25 μm, shown in Fig. 1a, is composed of a central sensitive area with a size of about 20 μm × 20 μm and two long legs which support the sensitive area. The sensitive area consists of multilayer films including a support layer made of 0.4 μm silicon nitride (Si_3_N_4_) film, thermal sensitive layer (VO_x_ thin film) with a thickness of 70 nm, and spiral-type antenna acting as THz absorption layer made of 0.05 μm aluminum (Al) thin film. A nickel–chromium (NiCr) thin film with a thickness of 0.2 μm is placed below the sensitive area as a reflection layer to form a resonant cavity 2 μm high for optimized absorption of IR radiation and thermal isolation of THz radiation. Spiral-type antenna structure is located on the Si_3_N_4_ support layer and limited with an outside diameter of 18 μm. Aimed at the size limitation of the support layer, in addition to traditional spiral-type antenna on the support layer shown in Fig. 1b, novel spiral-type antenna-coupled micro-bridge structures are proposed. Linear antennas are introduced and integrated on the bridge legs, which results in increased areas of the original spiral-type antennas on the support layer. Figure 1c–e shows spiral-type antennas with a single separate linear antenna, two separate linear antennas, and two connected linear antennas on the bridge legs, respectively.

### Spiral-Type Antenna on the Support Layer

Traditional spiral-type antenna-coupled micro-bridge structure, shown in Fig. 1b, was first studied with the antenna on the support layer. The structural parameters (indicated in Fig. 1b) of spiral-type antenna were optimized, and the influence of each parameter on THz absorption characteristics was discussed.

**Fig. 2:**
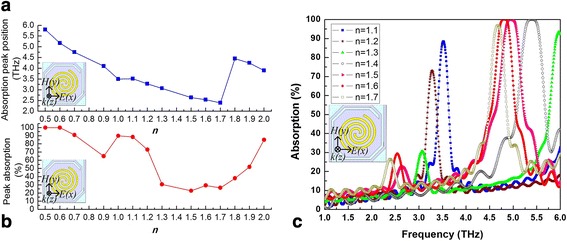
Variation curves of absorption peak position (**a**) and peak absorption rate (**b**) and THz wave absorption curves (**c**) of micro-bridge structures with different rotation angles (360*n) of spiral-type antennas on the support layer

For spiral-type antenna on the support layer with an antenna line width of 1 μm and a rotation angle (the rotation angle starting from the center of the antenna) of 360*n (*n* changes in 0.5~2.0), the variations of absorption peak position and peak absorption rate of antenna-coupled micro-bridge structures with *n* are shown in Fig. 2a, b, respectively.

It can be seen from Fig. 2a, b that the peak absorption frequency and peak absorption rate decrease when *n* increases from 0.5 to 0.9. The peak absorption rate decreases to 65% at 4.1 THz when *n* = 0.9 and then increases to 90% at 3.5 THz when *n* = 1. When *n* = 1~1.5, the peak absorption frequency and peak absorption rate continue to decrease with the increase of rotation angle. The peak absorption frequency decreases to 2.64 THz when *n* = 1.5; however, the peak absorption rate decreases to 22.8%. An absorption of 30% is obtained at 2.53 THz when *n* = 1.6. The minimum peak absorption frequency occurs at 2.39 THz when *n* = 1.7 and then the absorption frequency increases to 4.45 THz when *n* = 1.8. When *n* = 1.8~2, the peak absorption frequency decreases again while the peak absorption rate increases with the increase of rotation angle. Figure 2a suggests that the absorption frequency keeps decreasing with the increase of rotation angle in several different ranges including *n* = 0.5~1, *n* = 1.1~1.7, and *n* = 1.8~2. The peak absorption rate also keeps decreasing when *n* = 0.5~0.9, *n* = 1~1.5, and *n* = 1.6~1.7. Antennas with greater rotation angles (360*n) when *n* > 2 are not considered due to the size limitation of the support layer. THz wave absorption curves of micro-bridge structures are shown in Fig. 2c with different rotation angles (360*n, *n* = 1.1~1.7) of spiral-type antennas on the support layer. Each absorption curve has multiple absorption peaks along the frequency axis, and the absorption peak at the lowest frequency is used to plot Fig. 2a, b aiming at optimized absorption of 2.52 THz wave radiated by high power far infrared CO_2_ gas laser. Figure 2 indicates that an absorption peak is obtained near 2.52 THz when *n* = 1.6 with a low absorption rate of 30%.

**Fig. 3:**
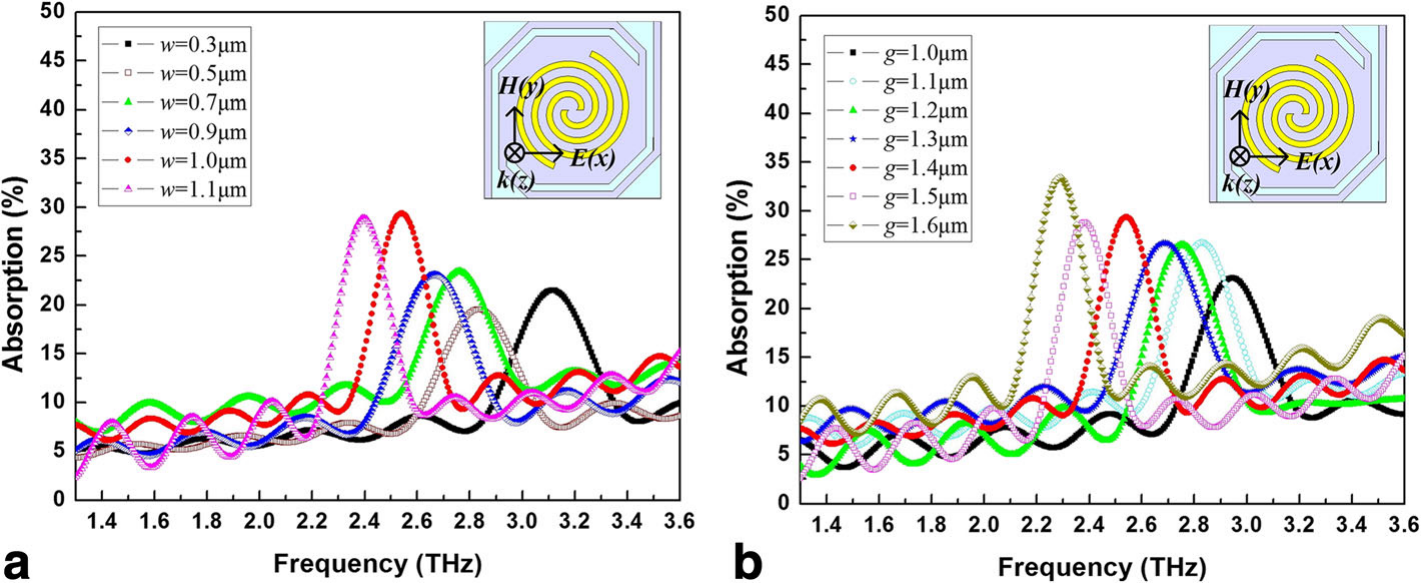
THz wave absorption curves of micro-bridge structures with spiral-type antenna on the support layer when *n* = 1.6 with different line width (**a**) and different spacing (**b**)

Figure 3a, b shows THz wave absorption curves of micro-bridge structures with spiral-type antenna on the support layer when *n* = 1.6 with different line width (*w*) and spacing (*g*), respectively. It can be seen that the peak absorption frequency decreases significantly, while the peak absorption rate increases slowly with the increase of line width and spacing. A similar conclusion is obtained when *n* = 1.1. The increases of line width and spacing lead to an increased size of the antenna. It seems that the increase of antenna area is propitious to reduce the absorption frequency but it does not contribute much to the absorption rate.

A more poor absorption near 2.52 THz is obtained for spiral-type antenna-coupled micro-bridge structure with a pixel size of 25 μm × 25 μm due to a smaller pixel size compared to 35 μm × 35 μm pixel structure reported in [29] which provides a higher absorption rate of 45% at 2.77 THz when *n* = 1.1 and 46% at 2.99 THz when *n* = 2.1. As we have previously concluded, to increase antenna area is an effective way for absorption frequency modulation, but it is limited by the size of the support layer and it becomes more severe for 25 μm × 25 μm pixel.

### Spiral-Type Antenna with a Single Separate Linear Antenna on One of the Bridge Legs

The legs of micro-bridge structure play the roles of mechanical support and electrical and thermal channels. Long bridge legs can provide low thermal conductivity and enhance the thermal insulation performance of the micro-bridge structure. However, it also reduces the effective size of the sensitive area, limiting the size of the absorbing film or structures. In order to achieve high absorption rate at lower frequency, linear antennas are introduced on the bridge legs for an increased area of the antennas. Figure 1c shows a spiral-type antenna with a single separate linear antenna on one of the bridge legs.

**Fig. 4:**
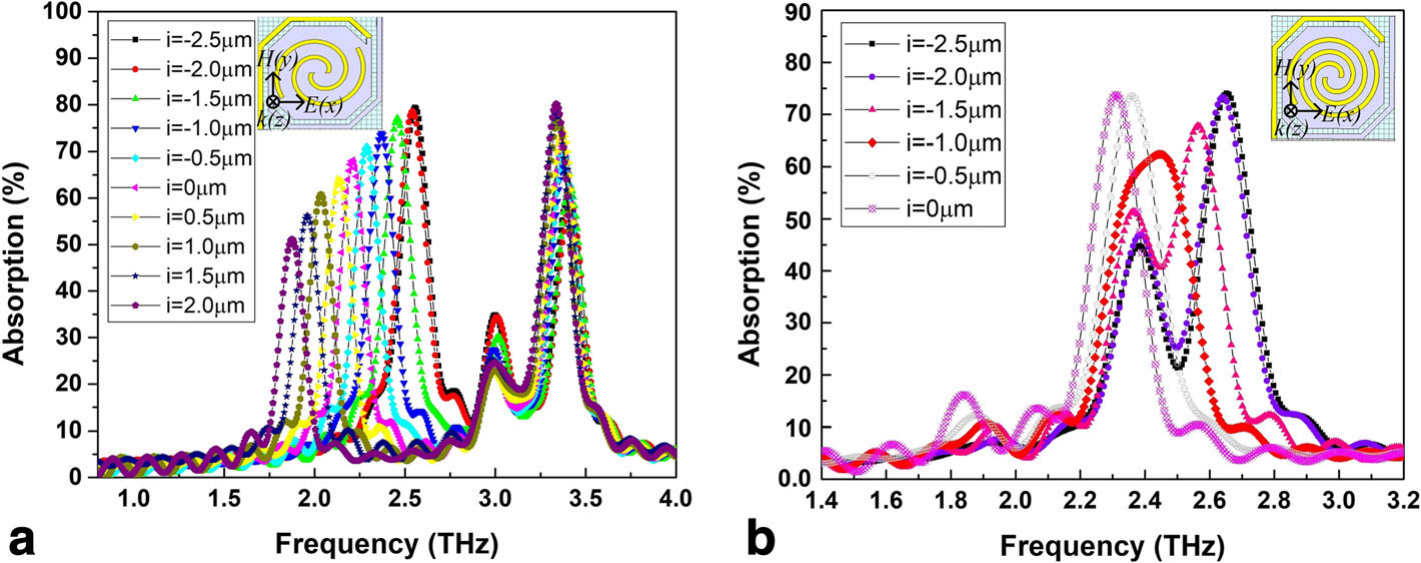
THz wave absorption curves of spiral-type antenna-coupled micro-bridge structures with a single separate linear antenna on one of the bridge legs when *n* = 1.1 (**a**) and *n* = 1.6 (**b**) for different linear antenna positions

Our research indicated that the port of the linear antenna on the bridge leg near the sensitive area side had a strong coupling absorption effect. So we set the rotation angle to 360*n (*n* = 1.1 and *n* = 1.6), the line width of the antenna to 1 μm, and the spacing to 2.5 μm (*n* = 1.1) and 1.4 μm (*n* = 1.6) and adjusted the distance (*i*, indicated in Fig. 1c with a partial enlarged drawing) between the antenna port on the bridge leg and the connection between the bridge leg and the sensitive area. THz wave absorption curves of spiral-type antenna-coupled micro-bridge structures with a single separate linear antenna on one of the bridge legs for different linear antenna positions when *n* = 1.1 and *n* = 1.6 are shown in Fig. 4a, b, respectively.

As shown in Fig. 4a, a new absorption peak appears at lower frequency when the antenna on the bridge leg is introduced, in addition to the original absorption peak near 3.5 THz. As the antenna port on the bridge leg gets close to the sensitive area (*i* changes from − 2.5 to 2 μm), the absorption at higher frequency remains roughly the same, while the peak absorption rate and the absorption frequency decrease at lower frequency. It becomes clear that the antenna on the bridge leg contributes to the absorption at lower frequency. The absorption curves of spiral-type antennas with a single separate linear antenna when *n* = 1.6, shown in Fig. 4b, indicate a wide absorption peak near 2.52 THz. This is because the absorption peaks of spiral-type antenna on the support layer and that of the antenna on the bridge leg occur in the close position. As *i* changes from − 2.5 to − 1 μm, the two absorption peaks get close to each other and broaden the absorption band. A wide absorption of more than 40% can be obtained in a bandwidth of 0.4 THz when *i* = − 1.5 and a single wide absorption peak is achieved with a half peak width of 0.3 THz when *i* = − 1.

### Spiral-Type Antenna with Two Separate Linear Antennas on the Bridge Legs

**Fig. 5:**
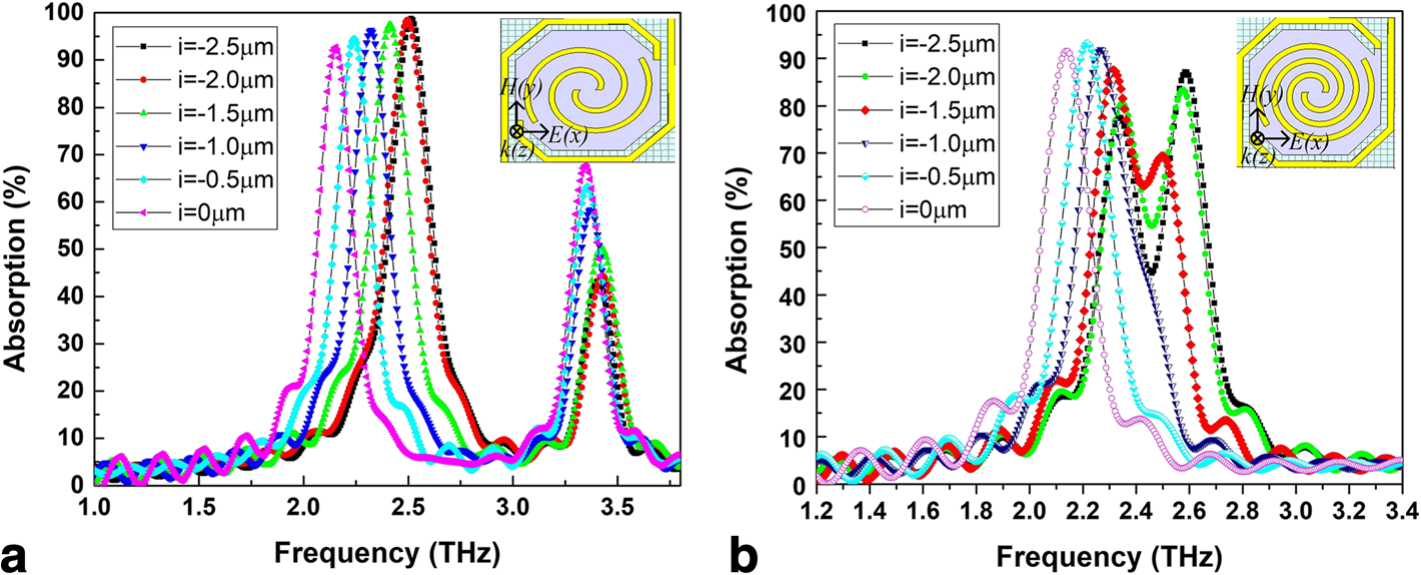
THz wave absorption curves of spiral-type antenna-coupled micro-bridge structures with two separate linear antennas on the bridge legs when *n* = 1.1 (**a**) and *n* = 1.6 (**b**) for different linear antenna positions

For spiral-type antenna with two separate linear antennas, shown in Fig. 1d, THz wave absorption curves of spiral-type antenna-coupled micro-bridge structures for different linear antenna positions when *n* = 1.1 and *n* = 1.6, with the same settings of other structure parameters including line width and spacing, are shown in Fig. 5a, b, respectively. The variations of THz absorption have the same tendency in general as that of spiral-type antenna with a single separate linear antenna shown in Fig. 4. The two legs of the micro-bridge structure are both used to prepare antennas on, so the area of the antenna is further enlarged. This results in much higher absorption rate (more than 90%) at lower frequency when *n* = 1.1 as shown in Fig. 5a compared to that of spiral-type antenna with a single separate linear antenna. The introduction of antennas on bridge legs also increases the absorption at original higher frequency. Wide absorption peaks are also obtained in Fig. 5b when *n* = 1.6 and the absorptions are enhanced significantly. It can be concluded that spiral-type antenna with two separate linear antennas on the bridge legs when *n* = 1.6 is more suitable to be used in THz micro-bolometer FPAs based on micro-bridge structures due to its higher absorption in wider band.

**Fig. 6:**
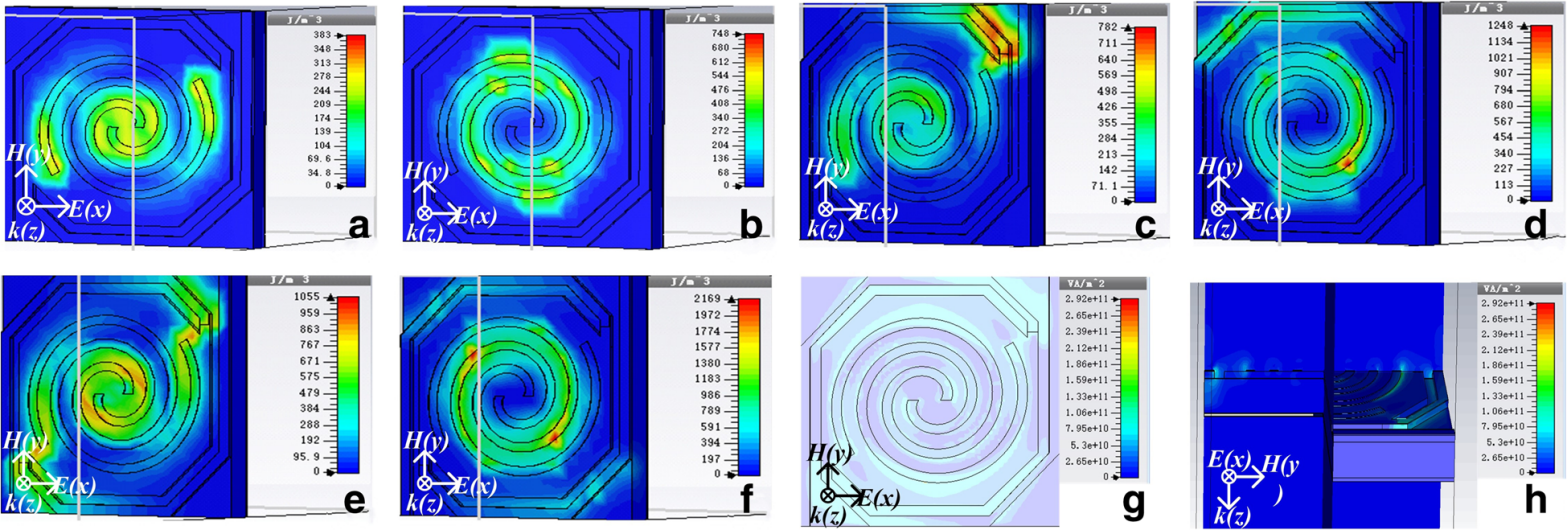
Distribution of electric field energy density, magnetic field energy density, and power loss. Energy density diagrams of electric field (**a**) and magnetic field (**b**) for spiral-type antenna on the support layer when *n* = 1.6; energy density diagrams of electric field (**c**) and magnetic field (**d**) for spiral-type antenna with a single separate linear antenna when *n* = 1.6 and *i* = − 2; energy density diagrams of electric field (**e**) and magnetic field (**f**) for spiral-type antenna with two separate linear antennas when *n* = 1.6 and *i* = − 2; power loss distribution in the micro-bridge structure coupled with spiral-type antenna with two separate linear antennas when *n* = 1.6 and *i* = − 2 from top view (**g**) and side view (**h**)

**Fig. 7:**
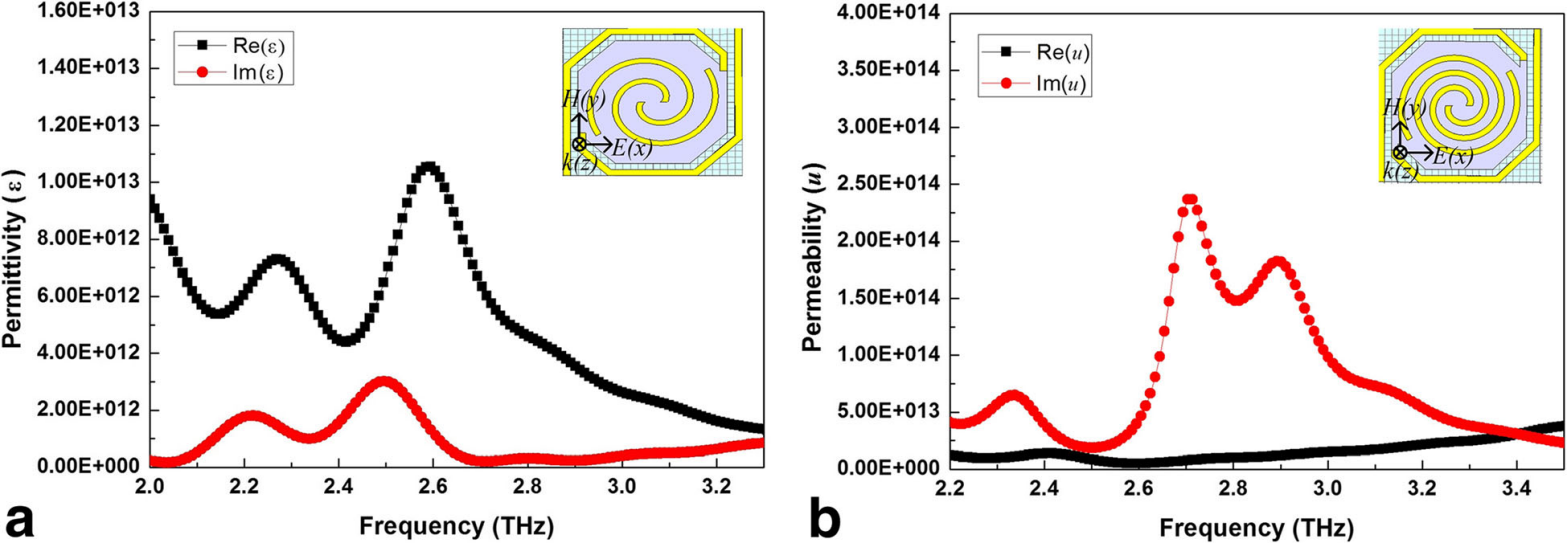
Real and imaginary parts of effective permeability (**a**) and permittivity (**b**) as a function of frequency for micro-bridge structure coupled with spiral-type antenna with two separate linear antennas when *n* = 1.6 and *i* = − 2

Figure 6 shows the energy density diagrams of electric field and magnetic field for the three kinds of spiral-type antenna-coupled micro-bridge structures designed before. It can be seen from Fig. 6a, b that for spiral-type antenna on the support layer, the absorption of electric field energy occurs mainly at the center and both ends of the spiral-type antenna while the antenna line contributes most of the absorption of magnetic field energy, which agrees with our earlier studies reported in [29]. Figure 6c, d shows that a strong coupling absorption effect of electric field energy occurs at the port of the single separate linear antenna on the bridge leg near the sensitive area side, and the antenna on the leg also contributes to the absorption of magnetic field energy. Similar phenomena can be observed for spiral-type antenna with two separate linear antennas on the bridge legs as shown in Fig. 6e, f. The absorption of both electric field energy and magnetic field energy is increased in absorption area and enhanced in absorption intensity due to enlarged antenna area. Figure 6g, h shows the power loss distribution in the micro-bridge structure coupled with spiral-type antenna with two separate linear antennas on the bridge legs when *n* = 1.6 and *i* = − 2 from top view and side view, respectively. It can be seen clearly from Fig. 6h that the power loss is confined almost entirely in the central sensitive area, which is propitious to cause temperature rise of thermosensitive VO_x_ thin film integrated in the central sensitive area. Power loss induced by central spiral-type antenna mainly occurs in the antenna layer while most of the loss caused by separate linear antennas on the bridge legs occurs in the Si_3_N_4_ support layer. This means that the absorption peak at higher frequency in Fig. 5a is caused by ohmic loss of central spiral-type antenna while the absorption peak at lower frequency is attributed to separate linear antennas on the bridge legs due to dielectric loss, which contributes to form a wide absorption peak as shown in Fig. 5b. Based on the transmission and reflection coefficients (*S* parameters) of the structure, the scattering data can be inverted to determine refractive index (*n*) and impedance (*z*), from which self-consistent values for effective permittivity (*ε*) and permeability (*μ*) can be obtained [30]. Figure 7a, b shows the real and imaginary parts of effective permeability and permittivity as a function of frequency for the micro-bridge structure coupled with spiral-type antenna with two separate linear antennas when *n* = 1.6 and *i* = − 2, respectively. It can be seen from Fig. 7 that obvious resonances occur around 2.52 THz, which induce the loss of THz radiation and the two absorption peaks as shown in Fig. 5b.

### Spiral-Type Antenna with Two Connected Linear Antennas on the Bridge Legs

**Fig. 8:**
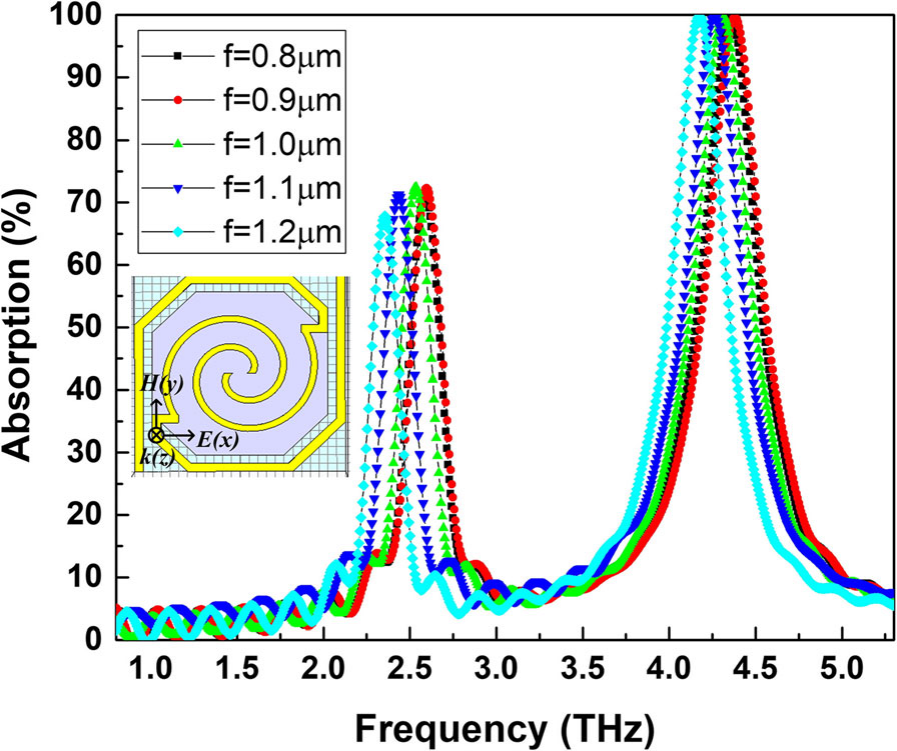
THz wave absorption curves of spiral-type antenna-coupled micro-bridge structures with two connected linear antennas on the bridge legs for different line width (*f*)

Another kind of spiral-type antenna, shown in Fig. 1e, was proposed with two connected linear antennas on the bridge legs. Figure 8 shows THz wave absorption curves of spiral-type antenna-coupled micro-bridge structures when *n* = 1.6, *g* (spacing) = 1.4 μm for different line width (*f*). Two apparent absorption peaks are observed in Fig. 8. The peak absorption position moves slowly to lower frequency with the increase of antenna line width, while the peak absorption rate changes little. An absorption of about 70% is obtained at 2.52 THz when *f* = 1 μm, and the absorption rate of each curve at 2.52 THz when *f* = 0.8~ 1.1 μm is above 50%. This indicates that the width difference of the antenna line which may be caused by the fabrication process has little influence on THz absorption, which is conducive to the design of spiral-type antenna-coupled micro-bridge structures and reduces the difficulty of manufacturing and realization of the designed structures for greater redundancy is allowed.

**Fig. 9:**
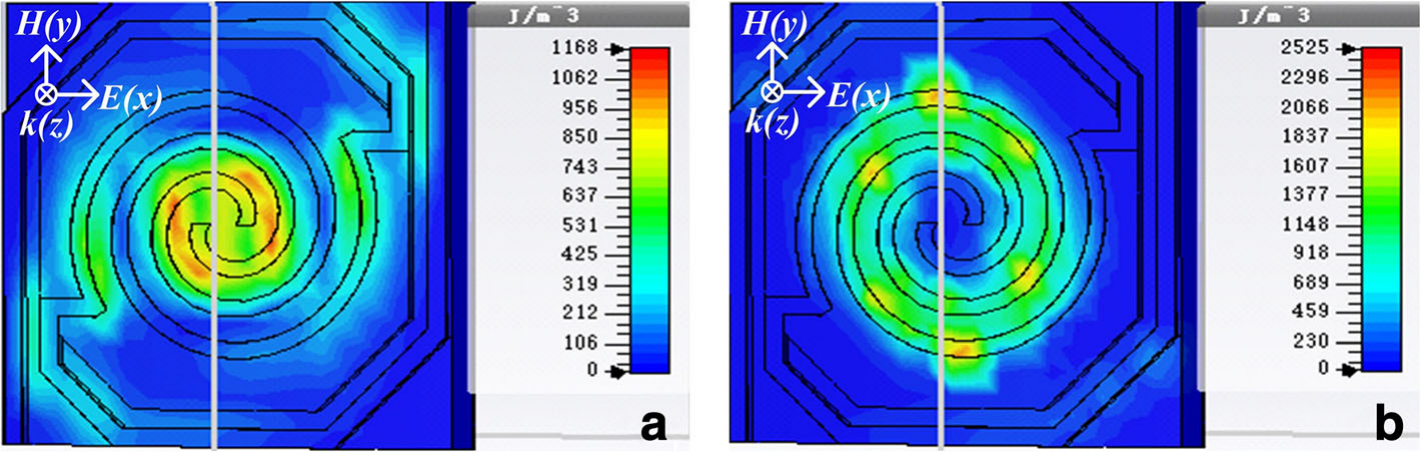
Energy density diagrams of electric field (**a**) and magnetic field (**b**) for spiral-type antenna-coupled micro-bridge structures with two connected linear antennas on the bridge legs and a line width of 1 μm

Figure 9 shows the energy density diagrams of electric field and magnetic field for spiral-type antenna with two connected linear antennas on the bridge legs when the line width is 1 μm. The absorption area of electric field energy, shown in Fig. 9a, mainly occurs in the sensitive area and the connection area between the bridge legs and the sensitive area. The absorption of magnetic field energy, shown in Fig. 9b, is mainly attributed to the contribution of the antenna on the support layer. Most of the absorption occurs on the support layer and can be transformed to temperature rise of the VO_x_ thin film.

The design of spiral-type antenna with two separate linear antennas or two connected linear antennas on the bridge legs, shown in Fig. 1d, e, is a good solution for high absorption rate at low absorption frequency of 2.52 THz when the rotation angle is set to 360*n (*n* = 1.6). The spiral-type antenna with two separate linear antennas provides wide absorption peak near 2.52 THz, while the spiral-type antenna with two connected linear antennas has a relatively stable absorption peak with the changing of the antenna line width. Another advantage of spiral-type antenna with two connected linear antennas is that the antenna can act as electrode lead for high integration and process simplification since the antenna and electrode lead layer can be fabricated by a single step photolithography and pattern process. This provides a highly integrated spiral-type antenna-coupled micro-bridge structure with high absorption at 2.52 THz and a highly compatible, process-simplified way to realize the structure.

For THz detector with antenna-coupled micro-bridge structure, the thermal response time (*τ*) depends on its effective thermal conductance (*G*_eff_) and total heat capacity (*C*_tot_) via *τ* = *C*_tot_/*G*_eff_. *G*_eff_ is defined via *G*_eff_ = *G*_leg_ − *αV*_bias_*I*_0_, where *α* is the temperature coefficient of current and *V*_bias_ and *I*_0_ are the bias voltage and current of the detector [31], respectively. *G*_leg_ = 2*σ*_th_*A*/*l* is the thermal conductance of the bridge legs, where *σ*_th_ is the leg thermal conductivity and *A* and *l* are the cross-sectional area and length of the bridge legs, respectively. It is multiplied by 2 for there are two legs. For a definite micro-bridge structure, heat conduction of the bridge legs is fixed; *G*_eff_ would also be fixed [32]. *τ* will be determined by *C*_tot_, which is the total heat capacity of the antenna and micro-bridge structure including the load such that *C*_tot_ = *C*_ant_ + *C*_bridge_. The heat capacity of the antenna is defined via *C*_ant_ = *c*_ant_*ρ*_ant_*V*_ant_, where *c*_ant_ is the antenna specific heat, *ρ*_ant_ is the antenna mass density, and *V*_ant_ is the antenna volume. *C*_bridge_ is defined in a similar manner to *C*_ant_. It can be concluded that *C*_tot_ is mainly restricted by antenna volume (*V*_ant_) for a definite antenna material on a fixed micro-bridge structure. That is why we expect to reduce antenna volume by using linear antennas rather than planar antennas to achieve lower thermal response time. For the antenna-coupled micro-bridge structure designed in this paper with a single metal layer acting as both the antenna and electrode lead layer, the total heat capacity is further reduced for *C*_tot_ ≈ *C*_bridge_. Assuming that the central sensitive area of a micro-bridge structure consists of Si_3_N_4_ film with a size of about 20 μm × 20 μm and a thickness of 0.4 μm, and the antenna layer is made of Al thin film with a thickness of 0.05 μm and covers 1/3 of the sensitive area, the heat capacity of Si_3_N_4_ film and the Al antenna can be calculated since the specific heat capacity and mass density of PECVD Si_3_N_4_ film are 0.17 J/(g*K) and 2500 Kg/m^3^, while that of Al thin film are 0.91 J/(g*K) and 2700 Kg/m^3^, respectively. The results suggest that for the antenna-coupled micro-bridge structure with a single antenna and electrode lead layer, the total heat capacity can be reduced to 83.7% of the traditional micro-bridge structure with two metal layers acting as the antenna and electrode lead layer separately, and the thermal response time can be reduced by 16.3% under the same thermal conductivity of the micro-bridge structure. This provides the potentiality of applications in high-performance THz micro-bolometer detectors with fast response.

## Conclusions

In this paper, we have carried out the design, simulation, and optimization of four kinds of spiral-type antenna-coupled micro-bolometers for THz applications in sensing and imaging. Compared to traditional spiral-type antenna on the support layer of micro-bridge structure, antennas are proposed with a single separate linear antenna, two separate linear antennas, or two connected linear antennas on the bridge legs. The structural parameters of spiral-type antenna are optimized and the influence of each parameter on absorption characteristics is discussed. The antenna area is enlarged and the absorption frequency is decreased due to the introduction of linear antennas on bridge legs. The spiral-type antenna with two separate linear antennas provides wide absorption peak near 2.52 THz, while the spiral-type antenna with two connected linear antennas has a relatively stable absorption peak with the changing of the antenna line width and provides possibility for high integration and process simplification of the micro-bridge structure. This paper presents the applications of spiral-type antennas in THz detector based on micro-bridge structure and discusses their advantages in THz absorption enhancement, absorption frequency modulation, response time improvement, and manufacturing process simplification.

## Methods

We performed finite-element numerical simulations using CST Microwave Studio 2016. We simulated a single cubic unit cell with a unit size of 25 μm × 25 μm as shown in Fig. 1f, with the antenna-coupled micro-bridge structure located at the center. The wave vector *k* propagated through the *z* direction with perfect electric field in *x*-*z* plane and perfect magnetic field in *y*-*z* plane. We set the input and output ports on the top and bottom faces of the cubic unit cell in the vacuum which are indicated as port “1” and port “2” in Fig. 1f, respectively. The simulation produced the frequency-dependent complex *S* parameters, from which we obtained the reflectance *R* = |*S*_11_|^2^ at port “1” and transmittance *T* = |*S*_21_|^2^ at port “2” with periodic boundary conditions (PBC) along the $\widehat{x}$ and $\widehat{y}$ directions. The absorptions of the antenna-coupled micro-bridge structures were calculated via *A* = 1 − |*S*_21_|^2^ − |*S*_11_|^2^. For the spiral-type antenna-coupled micro-bridge structures proposed in Fig. 1a–e, the Al and NiCr thin films were modeled as lossy metal with the default conductivity *σ*_Al_ = 3.56 × 10^7^ S/m and *σ*_NiCr_ = 1 × 10^7^ S/m. Si_3_N_4_ thin film was modeled as optical silicon nitride film with a dispersion permittivity *ε*_Si3N4_ of 2nd order model (fit) in CST and a permeability of 1. The resonant cavity was treated with *ε*_vacuum_ = 1 and *σ*_vacuum_ = 0 S/m.

## Abbreviations

FET: Field effect transistors
FPA: Focal plane array
IR: Infrared
MOSFET: Metal-oxide-semiconductor FET
NiCr: Nickel–chromium
PBC: Periodic boundary conditions
QCL: Quantum cascade lasers
QW: Quantum well
Si_3_N_4_: Silicon nitride
SIS: Superconductor–insulator–superconductor tunnel junction
THz: Terahertz
VO_x_: Vanadium oxide
